# Adverse Effects on the Thyroid of Chinese Pregnant Women Exposed to Long-Term Iodine Excess: Optimal and Safe Tolerable Upper Intake Levels of Iodine

**DOI:** 10.3390/nu15071635

**Published:** 2023-03-28

**Authors:** Wen Wu, Wenxing Guo, Naifan Zhang, Min Gao, Kexin Zhang, Elizabeth N. Pearce, Shaohan Li, Zhiyuan Ren, Ying Yang, Chunxi Wang, Wanqi Zhang

**Affiliations:** 1 Department of Nutrition and Food Hygiene, School of Public Health, Tianjin Medical University, Tianjin 300070, China; 2 Department of Nutrition and Food Hygiene, School of Public Health, Capital Medical University, Beijing 100069, China; 3 Section of Endocrinology, Diabetes, and Nutrition, School of Medicine, Boston University, Boston, MA 02118, USA; 4 Tianjin Key Laboratory of Environment, Nutrition and Public Health, Center for International Collaborative Research on Environment, Nutrition and Public Health, Tianjin 300070, China; 5 Department of Endocrinology and Metabolism, Tianjin Medical University General Hospital, Tianjin 300070, China

**Keywords:** pregnant women, tolerable upper intake levels, urinary iodine excretion, thyroid dysfunction, iodine

## Abstract

Ensuring optimal iodine nutrition in pregnant women is a global public health concern. However, there is no direct data on safe tolerable upper intake levels (ULs) for pregnant women. A cross-sectional study was performed to determine the ULs of pregnant women. A total of 744 pregnant women were enrolled in this study. The median (IQR) urinary iodine concentration (UIC) in pregnant women was 150.2 (87.6, 268.0) μg/L, and the urinary iodine excretion (UIE) over 24 h was 204.2 (116.0, 387.0) μg/day. Compared with those with a UIE figure of between 150–250 μg/day, the reference group, the prevalence of thyroid dysfunction was 5.7 times higher (95%CI: 1.7, 19.2) in pregnant women with a UIE figure of between 450–550 μg/day, and 3.9 times higher (95%CI: 1.5, 10.3) in pregnant women with a UIE figure of ≥550 μg/day. Compared with an estimated iodine intake (EII) of between 100–200 μg/day, the reference group, the prevalence of thyroid dysfunction was 4.3 times higher (95%CI: 1.3, 14.4) in pregnant women with a UIE figure of between 500–600 μg/day, and 3.6 times higher (95%CI: 1.5, 8.9) in pregnant women with UIE of ≥600 μg/day. In general, our cross-sectional study found that excessive iodine intake during pregnancy appears to directly increase the risk of thyroid dysfunction. Avoiding chronic iodine intakes of 500 μg/day or higher or having a UIE figure of ≥450 μg/day is recommended for pregnant women in China.

## 1. Introduction

As an essential trace element, iodine plays an important role in the synthesis of thyroid hormones [1,2]. Iodine status has long been regarded as an environmental determinant for thyroid dysfunction; excessive or deficient iodine has adverse health effects, with a U-shaped relationship between human health and iodine intake [3]. Iodine deficiency in pregnant women may lead to hypothyroidism, miscarriage, neonatal development retardation, and infant death, and iodine excess may result in goiter, thyroid function disorders, and thyroiditis [4]. Our previous studies have indicated that excessive iodine intake has adverse effects on thyroid function in pregnant women and infants [5,6]. Adequate iodine nutrition in pregnant women is essential to maintain their thyroid health and the thyroid hormones for fetal neurodevelopment [7]. Ensuring optimal iodine nutrition in pregnant women is a global public health imperative. Because of the successful implementation of universal salt iodization programs in many parts of the world, iodine deficiency disorders have become less frequent. The public health concern in some regions has gradually shifted toward risks related to iodine excess rather than deficiency.

For ensuring appropriate iodine status for pregnant women, the World Health Organization (WHO)/United Nations International Children’s Emergency Fund (UNICEF)/International Council for Control of Iodine Deficiency Disorders (ICCIDD) recommended the reference intervals of urinary iodine concentration (UIC) for pregnant women as follows: insufficient (<150 μg/L), adequate (150–249 μg/L), more than adequate (250–499 μg/L), and excessive (>500 μg/L) [8]. However, because of the high variability of urinary iodine, the above criteria are more suitable for evaluating the iodine nutritional status of the pregnant women at the population level. Dietary Reference Intakes (DRIs) are more commonly used to evaluate individual iodine nutritional status. The long-term iodine intake of pregnant women should not be more than the safe tolerable upper intake levels (ULs) to avoid possible health damage [4].

Many countries and organizations have established the ULs for pregnant women. WHO recommended a UL of 500 μg/day for pregnant women. In the European Union, a UL of 600 μg/day has been recommended in pregnancy [9]. In the United Kingdom, a UL of 1000 μg/day has been recommended in pregnancy [10]. A UL of 1100 μg/day has been recommended for United States, Canada, Australia, and New Zealand [11,12,13], and 2000 μg/day for pregnant women has been recommended in Japan [14]. The current UL for pregnant women in China has been defined as 600 μg/day, based on data from non-pregnant adults [15]. None of the above UL standards have been based on research in pregnant women.

Interventional studies evaluating thyroid function in pregnant women in response to variable doses of iodine have been inconclusive. Due to ethical constraints, interventional studies on pregnant women are not appropriate. This study was conducted in an area with a large variability in water iodine in the natural environment and a restricted iodized salt supply. We aimed to comprehensively assess changes in thyroid function in pregnant women with long-term exposure to a wide range of iodine intakes, in order to provide data to inform guidelines for safe levels of iodine intake during gestation.

## 2. Materials and Methods

### 2.1. Study Population

**Figure 1:**
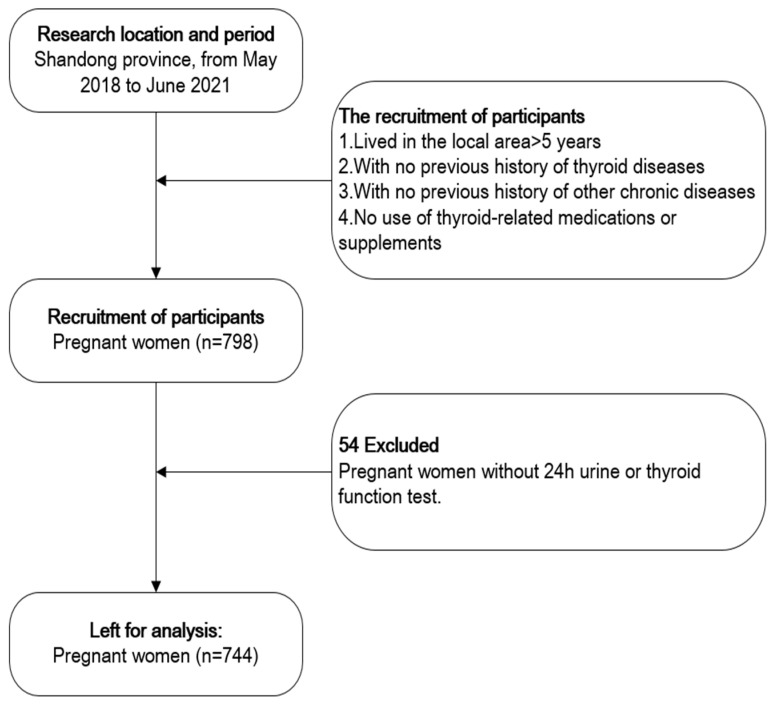
Flow chart of participant screening process.

A cross-sectional study was performed in Gaoqing County, Shandong province in China. Gaoqing is located in the lower reaches of the Yellow River. Historically, the flow of the Yellow River caused iodine deposition in the soil of the region, leading to excessive iodine intake from the high iodine content in the local drinking water. In 2009, the Chinese government ceased the supply of iodized salt in Gaoqing to prevent the possible health effects of excess iodine. Throughout our entire study, only non-iodized salt was available in Gaoqing. Laboratory testing confirmed that all pregnant women included in our study used non-iodized salt. After the implementation of an iodine reduction and water conversion project in 2014 in Gaoqing, some residents’ drinking water was changed from deep well water to Yellow River water, significantly reducing the water iodine concentration. However, there are also some residents whose drinking water sources remain unchanged. Therefore, Gaoqing has a significant volume of iodine concentration in drinking water. This study was performed between May 2018 and June 2021. Pregnant women who had lived in the local area for fewer than 5 years or who were using iodine-containing supplements were excluded. Pregnant women who came for routine maternity examinations were given a detailed explanation of this study and provided written informed consent. All procedures were approved by the Ethics Committee of Tianjin Medical University according to the Code of Ethics of the World Medical Association (Declaration of Helsinki). This information on the research has been registered on ClinicalTrials.gov (ID: NCT03710148, 15 October 2018). A total of 798 pregnant women were enrolled and 744 pregnant women were included in the final analysis (Figure 1).

### 2.2. Baseline Data Collection

All pregnant women presenting for their routine antenatal care during pregnancy were invited to participate in our study after a general description of the project. Study participants completed a questionnaire to provide information regarding ethnicity, age, pre-pregnancy weight, history of endocrine disease, and weeks of gestation. They also provided information about the source of drinking water as well as their education and employment. After the completion of the questionnaire, researchers would check the questionnaire filled by the participants with their health records, and if there were inconsistencies, researchers would immediately check with volunteers. Then, weight and height were measured with an accuracy of 0.1 kg and 0.01 cm, respectively. Body mass index (BMI) was calculated according to the following formula: BMI = weight in kilograms/height in square meters.

### 2.3. Urine Sample Collection and Analysis

Participants were instructed to empty their bladders before the collection of the urine samples over 24 h. Over the next 24 h, all urine was collected in 5-L polyethylene bottles. After the participants returned the polyethylene bottle the next day, the urine volume over 24 h was measured, and two 5-mL aliquots were taken from each bottle of urine. All of the urine samples were stored at −80 °C. The UIC was analyzed using inductively coupled plasma mass spectrometry (iCAP Q, Thermo Fisher Scientific, Frankfurt am Main, Germany) in the Tianjin Key Laboratory of Environmental Nutrition and Population Health, Tianjin Medical University. The urine iodine excretion (UIE) over 24 h was calculated by multiplying the UIC over 24 h by the urine volume. The UIE was categorized into 6 groups: <150 μg/day, 150–250 μg/day, 250–350 μg/day, 350–450 μg/day, 450–550 μg/day, and ≥550 μg/day.

### 2.4. Iodine Intake Estimation

Approximately 92% of the iodine ingested by the body is excreted via the urine [13,16], so we calculated the estimated iodine intake (EII) as EII (μg/day) = UIE (μg/day)/0.92. The EII was categorized as 6 groups: <100 μg/day, 100–200 μg/day, 200–300 μg/day, 300–400 μg/day, 400–500 μg/day, and ≥500 μg/day.

### 2.5. Blood Sample Collection and Analysis

After an overnight fast, blood samples were obtained between 8:00 and 11:00 a.m. Samples were centrifuged at 3000 r/min at room temperature. The serum was separated and stored frozen at −80 °C and sent to the Tianjin Maternal and Child Health Center for thyroid function testing within two weeks. Free thyroxine (FT4) and thyroid-stimulating hormone (TSH) were measured using chemiluminescence immunoassays (ADVIA Centaur CP, Siemens, Bayer Healthcare, Barmen, Germany). The lowest detection limits of TSH and FT4 were 0.008 mIU/L and 1.3, respectively. The intra-assay CVs (Coefficient of Variations) for serums TSH and FT4 were 2.1–4.9%, 1.7–4.2%, respectively, and the inter-assay CVs for serums TSH and FT4 were 1.5–4.4% and 1.4–3.1%, respectively. The reference ranges for serums TSH and FT4 were shown in Table 1. We defined thyroid dysfunction as any of the following thyroid diseases during pregnancy: overt hypothyroidism (elevated TSH levels and low FT4 levels), subclinical hypothyroidism (elevated TSH levels and normal FT4 levels), hypothyroxinemia (normal TSH levels and low FT4 levels), and thyrotoxicosis (low TSH levels and elevated FT4 levels).

### 2.6. Thyroid Volume Measurements and Thyroid Nodules

The thyroid volume was measured by a professional operator with the use of a HaiYing HY5511 ultrasound machine equipped with a 4-cm 7.5-MHz linear transducer. Before the study, the operator’s performance was validated against that of an expert from the Shandong Center of Disease Control. Measurements were performed with subjects sitting upright in a straight-back chair with the neck extended. For each thyroid lobe, the maximum width (*W*) was measured at the transverse section, the maximum length (*L*) and depth (*D*) were determined at the longitudinal section, and the number of thyroid nodules was recorded. The volume of each lobe was calculated with the use of the formula proposed by Brunn [17]: the thyroid volume = 0.479 (mL) × *W* (cm) × *L* (cm) × *D* (cm). The Tvol was the sum of both lobes (the isthmus was not included); a thyroid volume >18 mL was diagnosed as indicative of a goiter.

### 2.7. Statistical Analysis

All statistical analysis were performed via SPSS25.0 (IBM, Inc., New York, NY, USA) and Microsoft Excel (Win10 2016). The means ± SDs are used to describe the normally distributed continuous variables. Because the spots UIC, UIE, TSH and FT4 were not normally distributed, the median (IQR) was used. Differences in the prevalence of thyroid dysfunction and thyroid nodules, serum TSH levels, and thyroid volumes in women with different UIE and EII groups were compared using chi-square tests and Kruskal–Wallis tests. Logistic regression models, adjusted for BMI, age, and the trimester of pregnancy, were constructed to assess the relationships between UIE and EII and thyroid dysfunction during pregnancy. *p* < 0.05 was considered significant.

## 3. Results

### 3.1. Basic Information

After excluding 54 pregnant women with incomplete information (missing blood sample or urinary sample over 24 h), a total of 744 participants were included in the final analysis. After our laboratory tests, all pregnant women used non-iodized salt in this study. The subjects’ characteristics are presented in Table 2. The means ± SDs for the gestational week, age, and BMI during pregnancy were 22.5 ± 8.3 weeks, 30.5 ± 5.6 years, and 25.4 ± 4.1 kg/m^2^, respectively. The age and BMI of the pregnant women differed statistically in terms of the gestational age. The median (IQR) of UIC in pregnant women was 150.2 (87.6, 268.0) μg/L, indicating a sufficient iodine status based on current WHO/UNICEF/ICCIDD criteria. The median UIE over 24 h and TSH during pregnancy were 204.2 (116.0, 387.0) μg/day and 1.6 (1.0, 2.2) mIU/L, respectively. The UIC of the pregnant women gradually decreased throughout pregnancy; however, no significant difference was found in UIE between different pregnancy periods.

### 3.2. Thyroid Dysfunction in Relation to Participant Characteristics

**Figure 2:**
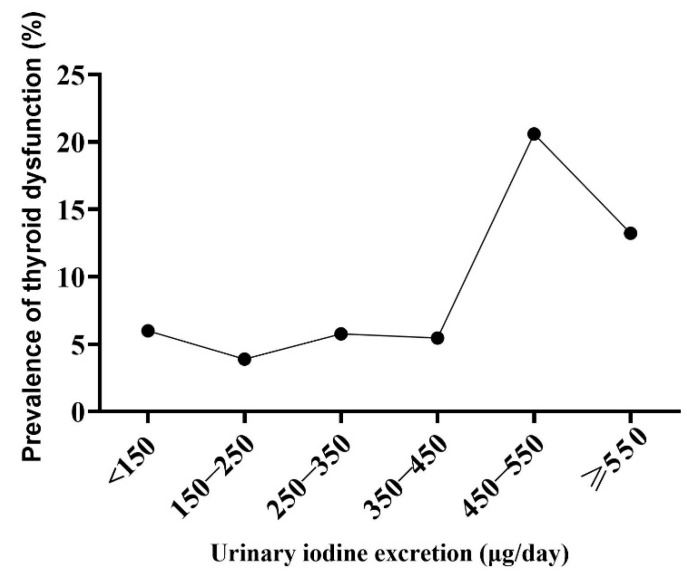
The prevalence of thyroid dysfunction in pregnant women among different urinary iodine excretion figures.

**Figure 3:**
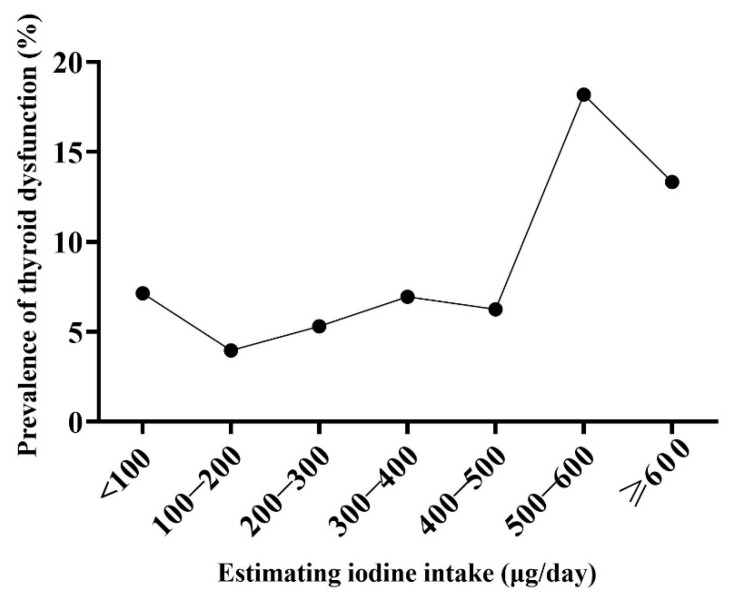
The prevalence of thyroid dysfunction in pregnant women among different estimating iodine intake figures.

Compared to the euthyroid women, the women with thyroid dysfunction did not differ by age, BMI, or gestational age (*p* > 0.05, not shown in table). As shown in Table 3, there were significant differences in hypothyroxinemia prevalence in terms of the UIE levels (*p* = 0.035), but no significant differences were found in subclinical hypothyroidism (*p* = 0.068) and the thyroid nodule (*p* = 0.484) prevalence. In this study, thyroid dysfunction referred to subclinical hypothyroidism and hypothyroxinemia. We found the lowest prevalence of thyroid dysfunction in pregnant women with a UIE figure of between 150–250 μg/day, and the prevalence increased in those with a UIE figure of between 450–550 μg/day or a UIE figure of ≥550 μg/day (*p* = 0.001). Similarly, we found the lowest prevalence of thyroid dysfunction in pregnant women with a EII figure of between 200–300 μg/day, and the prevalence increased in those with a UIE figure of between 500–600 μg/day or a UIE figure of ≥600 μg/day (*p* = 0.010). However, there were no significant differences in subclinical hypothyroidism, hypothyroxinemia, and the thyroid nodules in relation to the EII levels. (Table 4). The prevalence of thyroid dysfunction increased sharply in pregnant women with UIE ≥ 450 μg/day or EII ≥ 500 μg/day (Figure 2 and Figure 3).

### 3.3. The Relationship between UIE and Thyroid Dysfunction

The results of a logistic regression model examining UIE as a predictor of thyroid dysfunction, adjusted for age, BMI, and trimester, are shown in Table 5. Compared with 150–250 μg/day, the reference group, no significant difference in the prevalence of thyroid dysfunction was found in pregnant women with a UIE figure of <150 μg/day, between 250–350 μg/day, or between 350–450 μg/day. The prevalence of thyroid dysfunction was 5.7 times higher (95% CI: 1.7, 19.2) in pregnant women with a UIE figure between 450–550 μg/day, and 3.9 times higher (95% CI: 1.5, 10.3) in pregnant women with a UIE figure ≥550 μg/day than in the reference group. The logistic regression analysis of other UIE groups for thyroid dysfunction and the rates of thyroid dysfunction across different UIE levels were presented in the Supplementary Materials.

### 3.4. The Relationship between EII and Thyroid Dysfunction

The results of a logistic regression model examining EII as a predictor of thyroid dysfunction, adjusted for age, BMI, and trimester are shown in Table 5. Compared with 100–200 μg/day, the reference group, no significant difference in the prevalence of thyroid dysfunction was found in pregnant women with a EII figure of <100 μg/day, between 200–300 μg/day, between 300–400 μg/day, or between 400–500 μg/day. The prevalence of thyroid dysfunction was 4.3 times higher (95% CI: 1.3, 14.4) in pregnant women with a EII figure of between 500–600 μg/day, and 3.6 times higher (95% CI: 1.5, 8.9) in pregnant women with a EII figure of ≥600 μg/day.

## 4. Discussion

To our knowledge, this is the first study to assess for an iodine UL in pregnant women using the UIE over 24 h figures and thyroid dysfunction. Our study was conducted in pregnant women with long-term exposure to a wide range of iodine intakes due to the variability in the drinking water iodine levels and a restricted iodized salt supply. By taking advantage of this geographical variability, pregnant women with long-term exposure to deficient, adequate, and excessive iodine intakes could be recruited without any intervention. The iodine intakes of pregnant women in our study ranged from 44.5–1394.7 μg/day (2.5% to 97.5% quantile, estimated using UIE), covering most current UL standards, allowing us to derive an iodine UL for pregnancy.

Thyroid dysfunction during gestation, which is associated with an increased risk of adverse pregnancy and perinatal outcomes, may result from either iodine deficiency or excess [18]. In 1948, Wolff and Chaikoff showed that when plasma iodine concentrations were raised to a critical level, thyroid hormone synthesis and secretion was prevented by blocking thyroidal iodine organization [19]. After a few days of continued exposure to excessive iodine, there is an “escape” from the acute Wolff–Chaikoff effect, which is mediated by the downregulation of a sodium iodide symporter, which transports iodine into thyroid cells, and normal thyroid hormone production resumes. However, the ability to completely escape the acute Wolff–Chaikoff effect does not mature in the fetus until after 36 weeks of gestation, so fetal hypothyroidism may occur in the presence of an excessive iodine load, even if the mother’s thyroid function is maintained [20]. Even subclinical hypothyroidism during pregnancy can impair pregnancy outcomes and offspring development. According to a retrospective study by Casey et al., pregnant women with subclinical hypothyroidism had a 23 fold increased risk of adverse outcomes. In China, a study found that the incidence of spontaneous abortion in the subclinical hypothyroid group was 15.48%, which was significantly higher than the normal group (8.86%) [21]. Haddow et al. also found that the offspring of pregnant women with hypothyroidism or subclinical hypothyroidism had a 7-point reduction in intelligence at the age of 7–9 compared to the offspring of healthy pregnant women [22]. Our previous studies have shown that excessive iodine intake during pregnancy can lead to thyroid dysfunction in pregnant women [6] and elevated TSH in newborns [5]. Some studies found that high iodine exposure in pregnant women can lead to a lower birth weight [23] and poor neurodevelopment [24]. Therefore, excessively high iodine exposure may be particularly detrimental in the setting of gestation [25,26].

Approximately 92% of the iodine ingested by the body is excreted via the urine [13,16]. The UIC is a reliable biomarker that reflects the recent iodine intake. The WHO recommends the use of median UIC values to evaluate the iodine status in populations of children, adults, and pregnant women [8]. A spot UIC is most frequently used to assess the iodine nutrition in pregnant women in large-scale epidemiological investigations. However, due to the high diurnal and day-to-day variability of UIC levels, a spot UIC cannot be used as a biomarker to assess the chronic iodine intakes of individuals [27]. In order to estimate individual iodine intake, 10 to 12 repeated spot UIC measurements are required [28], which is logistically infeasible for large studies. In this study, we found that the UIC of pregnant women decreased throughout pregnancy periods, which is the same as the results of a previous study in China [29]. However, no significant difference was found in the UIE of pregnant women between different pregnancy periods. The spot urine and UIC was affected by urine dilution in pregnant women, but the urine and UIE over 24 h figures are not affected by urine dilution, which was the possible reason for the phenomenon. Previously, we defined the iodine UL of school-aged children based on UIE over 24 h, which was demonstrated to be more accurate and reproducible than spot UIC for estimating the individual iodine intake in large population-based cross-sectional surveys [3,30,31]. Perrine et al. suggested that 24-h urine collection is most reliable for the determination of iodine status [32]. Therefore, we used UIE as a biomarker for iodine nutrition evaluation in pregnant women to estimate the EII during pregnancy.

The WHO recommend that a daily intake greater than 500 μg/day is not necessary as it would not provide any additional benefit for health and may theoretically be associated with impaired thyroid function; however, the scientific evidence for this is weak [33]. Our study found the lowest prevalence of thyroid dysfunction in pregnant women with a UIE figure of between 150–250 μg/day or a EII of between 100–200 μg/day. The risk of thyroid dysfunction was significantly increased in pregnant women with a UIE figure of ≥450 μg/day or a EII of ≥500 μg/day. Based on our result, a UL of <500 μg/day for pregnant women is recommended, which is lower than the current UL standard of 600 μg/day in China [15] and the 1100 μg/day standard recommended in the United States [11]. Compared to prior studies, our results may be more robust because the data that were obtained in pregnant women exposed a wide range of iodine intakes over the long term.

Teng et al. suggested that the UIC of pregnant women should not exceed 250 μg/L, because this was associated with a significantly high risk of subclinical hypothyroidism, which is similar to the findings from our previous investigations in areas with high iodine [34]. These indicated that in both iodine-suitable and high iodine areas of China, a UIC ≥ 250 μg/L is a risk factor for thyroid dysfunction of pregnant women. The median urine volume over 24 h of pregnant women in this study was 1.74 L. Based on the UIC < 250 μg/L, the estimated UIE over 24 h should be less than 437.5 μg/day, similar to the conclusion in our study: UIE should be less than 450 μg/day.

Our study had some limitations. First, the iodine intake was not calculated directly via the measurement of dietary intakes or metabolic experiments, but was estimated using the UIE figure recorded over 24 h. Second, due to the cross-sectional study design, we could not establish the causality between iodine intake and thyroid dysfunction.

## 5. Conclusions

In general, excessive iodine intake during pregnancy appears to directly increase the risk of thyroid dysfunction. Therefore, avoiding chronic iodine intakes of 500 μg/day or higher or having a UIE figure of ≥450 μg/day is recommended for pregnant women in China.

## Figures and Tables

**Table 1:** Reference ranges for serum TSH and FT4 during pregnancy in Chinese women.

Indicators	1st Trimester	2nd Trimester	3rd Trimester
TSH	0.03–4.51 mIU/L	0.05–4.50 mIU/L	0.47–4.54 mIU/L
FT4	11.8–21.00 pmol/L	10.6–17.60 pmol/L	9.2–16.70 pmol/L

TSH: Thyroid-stimulating hormone; FT4: free thyroxine.

**Table 2:** Characteristics of pregnant women (N = 744).

Variables	1st Trimester	2nd Trimester	3rd Trimester	Total	*p*
n	80	449	215	744	-
Age (years)	29.6 ± 5.2	30.9 ± 5.6	30.1 ± 5.7	30.5 ± 5.6	0.049 ^a^
BMI (kg/m^2^)	23.4 ± 4.3	24.9 ± 4.0	27.2 ± 3.5	25.4 ± 4.1	<0.001 ^a^
UIC (μg/L)	194 (100.8, 424.9)	145.8 (82.6, 256.3)	143.8 (90.5, 264.9)	150.2 (87.6, 268)	0.019 ^b^
UIE (μg/day)	240 (112.0, 491.5)	197.8 (114.0, 358.0)	207 (121.5, 426.1)	204.2 (116.0, 387)	0.143 ^b^
Tvol (mL)	9.2 (7.0, 10.4)	8.8 (6.8, 11.5)	8.8 (6.7, 10.9)	8.8 (6.8, 11.1)	0.492 ^b^
TSH (mIU/L)	1.5 (0.9, 2.2)	1.5 (1.0, 2.1)	1.7 (1.1, 2.4)	1.6 (1.0, 2.2)	0.026 ^b^
FT4 (pmol/L)	16.6 (15.1, 17.8)	14.9 (13.4, 16.3)	14.2 (13.1, 15.4)	14.8 (13.5, 16.2)	<0.001 ^b^

BMI, body mass index; UIE, urinary iodine excretion; UIC, urinary iodine concentration; Tvol, thyroid volume TSH, thyroid-stimulating hormone; FT3, free triiodothyronine; FT4, free thyroxine; ^a^ Analysis of variance (ANOVA); ^b^ Analysis using the Kruskal–Wallis test.

**Table 3:** Thyroid dysfunction and thyroid nodules in pregnant women in terms of UIE (N = 744).

UIE, μg/day	Subclinical Hypothyroidism, n (%)	Hypothyroxinemia, n (%)	Thyroid Dysfunction, n (%)	Thyroid Nodules, n (%)
<150	9 (3.4)	7 (2.6)	16 (6.0)	71 (27.5)
150–250	3 (1.7)	4 (2.2)	7 (3.9)	45 (25.4)
250–350	4 (4.6)	1 (1.1)	5 (5.7)	21 (25.0)
350–450	2 (3.6)	1 (1.8)	3 (5.5)	20 (37.7)
450–550	3 (8.8)	4 (11.8)	7 (20.6)	6 (19.4)
≥550	10 (8.3)	6 (5.0)	16 (13.2)	31 (26.5)
total	31 (4.2)	23 (3.1)	54 (7.3)	194 (26.9)
*p*	0.068	0.035	0.001	0.484

Analysis of chi-square test; thyroid dysfunction, including subclinical hypothyroidism and hypothyroxinemia. UIE, urinary iodine excretion.

**Table 4:** Thyroid dysfunction and thyroid nodules in pregnant women in terms of EII (N = 744).

EII, μg/day	Subclinical Hypothyroidism, n (%)	Hypothyroxinemia, n (%)	Thyroid Dysfunction, n (%)	Thyroid Nodules, n (%)
<100	4 (3.6)	4 (3.6)	8 (7.1)	30 (28.6)
100–200	5 (2.2)	4 (1.8)	9 (4.0)	59 (26.3)
200–300	4 (3)	3 (2.3)	7 (5.3)	35 (27.1)
300–400	3 (4.2)	2 (2.8)	5 (6.9)	17 (23.9)
400–500	2 (4.2)	1 (2.1)	3 (6.3)	17 (37.8)
500–600	3 (9.1)	3 (9.1)	6 (18.2)	6 (20.0)
≥600	10 (8.3)	6 (5.0)	16 (13.3)	30 (25.9)
total	31 (4.2)	23 (3.1)	54 (7.3)	194 (26.9)
*p*	0.128	0.292	0.01	0.677

Analysis of chi-square test; thyroid dysfunction, including subclinical hypothyroidism and hypothyroxinemia. EII, estimated iodine intake.

**Table 5:** Logistic regressions of UIE for thyroid dysfunction (N = 744).

Groups	Unadjusted		Adjusted *	
	OR	95% CI	OR	95% CI
Model 1: UIE (μg/day)				
<150	1.6	0.6–3.9	1.7	0.7–4.6
150–250	Ref	-	Ref	-
250–350	1.5	0.5–4.9	1.4	0.4–5.0
350–450	1.4	0.4–5.7	1.7	0.4–7.0
450–550	6.4	2.1–19.7	5.7	1.7–19.2
≥550	3.8	1.5–9.5	3.9	1.5–10.3
Model 2: EII (μg/day)				
<100	1.9	0.7–5.0	2.0	0.7–5.6
100–200	Ref	-	Ref	-
200–300	1.4	0.5–3.7	1.3	0.4–3.8
300–400	1.8	0.6–5.6	1.5	0.4–5.2
400–500	1.6	0.4–6.2	1.9	0.5–7.3
500–600	5.4	1.8–16.3	4.3	1.3–14.4
≥600	3.7	1.6–8.7	3.6	1.5–8.9

* Logistic regression was adjusted BMI, age, and trimester of pregnancy. UIE, urinary iodine excretion, EII, estimated iodine intake, BMI, body mass index.

## Data Availability

The data presented in this study are available on request from the corresponding author. The data are not publicly available due to confidentiality requirements for funding funds.

## Supplementary Materials

The following supporting information can be downloaded at: https://www.mdpi.com/article/10.3390/nu15071635/s1, Table S1: Logistic regressions of UIE for thyroid dysfunction (N = 744); Table S2: The rate of thyroid dysfunction with different UIE level.
