# Supplementary material for: Adverse Effects on the Thyroid of Chinese Pregnant Women Exposed to Long-Term Iodine Excess: Optimal and Safe Tolerable Upper Intake Levels of Iodine
Source: Nutrients. 2023 Mar 28;15(7):1635. doi: 10.3390/nu15071635 (PMC10097226; doi:10.3390/nu15071635)
Supplement: Supplementary file 1 [file nutrients-15-01635-s001.zip › nutrients-2250691-supplementary.pdf]

Supplementary Table S1. Logistic regressions of UIE for thyroid dysfunction (N = 744).

	Unadjusted		Adjusted *	
	OR	*95% CI*	*OR*	*95% CI*
Model 1: UIE (μg/day)				
<150	1.6	0.6–3.9	1.7	0.7–4.6
150–250	Ref	-	Ref	-
250–350	1.5	0.5–4.9	1.4	0.4–5.0
350–450	1.4	0.4–5.7	1.7	0.4–7.0
450–550	6.4	2.1–19.7	5.7	1.7–19.2
≥550	3.8	1.5–9.5	3.9	1.5–10.3
Model 2: UIE (μg/day)				
<100	1.9	0.7–5.1	2.3	0.8–6.3
100–200	Ref	-	Ref	-
200–300	1.7	0.6–4.7	1.5	0.5–4.6
300–400	1.9	0.5–6.4	2.2	0.6–7.8
400–500	4.5	1.5–13.6	4.3	1.3–14.3
500–600	4.4	1.2–15.7	4.7	1.3–17.4
≥600	4.4	1.8–10.8	4.5	1.8–11.6

* Logistic regression was adjusted BMI, age, and trimester of pregnancy.
UIE, urinary iodine excretion; BMI, body mass index.

Supplementary Table S2. the rate of thyroid dysfunction with different UIE level.

UIE (μg/d)	n	Number of Cases	Rate (%)
<50	28	4	14.29
50–100	110	5	4.55
100–150	129	7	5.43
150–200	99	1	1.01
200–250	81	6	7.41
250–300	54	2	3.70
300–350	33	3	9.09
350–400	30	1	3.33
400–450	25	2	8.00
450–500	18	4	22.22
500–550	16	3	18.75
550–600	13	1	7.69
⩾600	108	15	13.89

UIE, urinary iodine excretion.
